# Soil conditions are a more important determinant of microbial community composition and functional potential than neighboring plant diversity

**DOI:** 10.1016/j.isci.2024.110056

**Published:** 2024-05-22

**Authors:** Ziva Louisson, Maria J. Gutiérrez-Ginés, Matthew Taylor, Hannah L. Buckley, Syrie M. Hermans, Gavin Lear

**Affiliations:** 1 School of Biological Sciences, University of Auckland, 3a Symonds Street, Auckland 1010, New Zealand; 2 Institute of Environmental Science and Research Ltd., 27 Creyke Road, Ilam, Christchurch 8041, New Zealand; 3 Waikato Regional Council, 160 Ward St, Hamilton 3204, New Zealand; 4 School of Science, Auckland University of Technology, 34 St Paul Street, Auckland 1010, New Zealand

**Keywords:** Soil science, Microbiology, Plant biology, Plant ecology, Soil biology

## Abstract

Replanting is an important tool for ecological recovery. Management strategies, such as planting areas with monocultures or species mixtures, have implications for restoration success. We used 16S and ITS rRNA gene amplicon sequencing and shotgun metagenomics to assess how the diversity of neighboring tree species impacted soil bacterial and fungal communities, and their functional potential, within the root zone of mānuka (*Leptospermum scoparium*) trees. We compared data from monoculture and mixed tree species plots and confirmed that soil microbial taxonomic and functional community profiles significantly differed (*p* < 0.001). Compared to the diversity of neighboring tree species within the plot, soil environmental conditions and geographic distance was more important for structuring the microbial communities. The bacterial communities appeared more impacted by soil conditions, while the fungal communities displayed stronger spatial structuring, possibly due to wider bacterial dispersal. The different mechanisms structuring bacterial and fungal communities could have implications for ecological restoration outcomes.

## Highlights

• Soil condition and distance structure microbial communities more than vegetation type
• Fungal communities may be more dispersal limited than bacterial communities
• Monocultural tree stands may have a role in reducing certain energy metabolism pathways

## Graphical abstract

**Figure:**
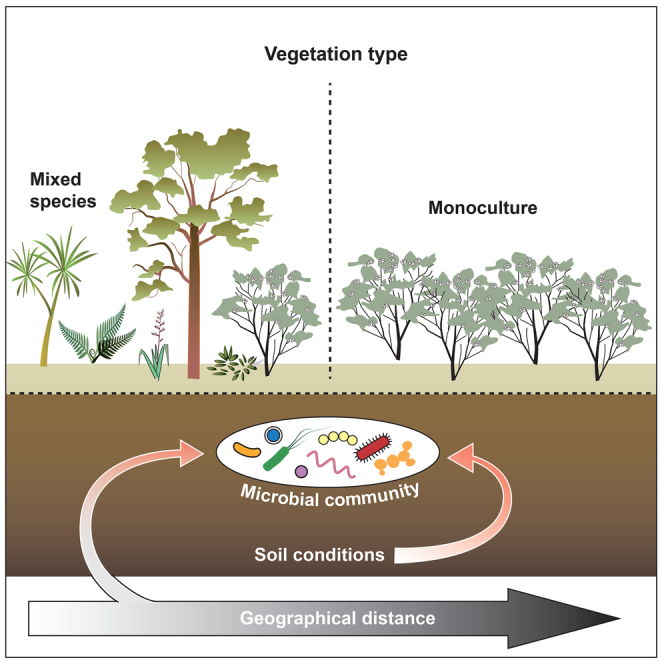

## Introduction

The co-evolution of plants and soil microorganisms has created tight, and often complex, associations between plants and belowground microbial communities.^1^ Soil microbes provide a suite of ecosystem services that can, directly and indirectly, promote plant health and diversity, for example, by producing growth-promoting phytohormones^2^ or increasing nutrient availability.^3^ Concurrently, greater plant diversity is suggested to support increased microbial diversity,^4^ with plants releasing exudates and organic nutrients in the form of detritus into the soil, which varies in quality among plant species.^5^ Some studies suggest that reduced plant diversity is associated with reductions in microbial diversity.^6^^,^^7^^,^^8^ However, others find no meaningful relationships between plant and soil microbial diversity^9^^,^^10^ or indicate that a relationship exists for certain taxonomic groups and not others. For example, Millard and Singh^11^ reported that bacterial diversity was not directly impacted by grassland plant diversity, but that fungal communities were. Differences in microbial biogeographic patterns can be driven by various factors such as soil conditions,^12^ soil depth,^13^ or host plants.^14^ These factors may have differing effects on bacterial and fungal communities due to their varying sensitivity to soil conditions^15^ or differing dependencies on plant products.^11^ Soil environmental conditions may account for inconsistent observations being reported from different studies; for example, reduced plant diversity may have a larger impact on the microbial diversity of lower quality soils.^6^ Additionally, legacy effects of previous land use can be a more important determinant of microbial community composition than vegetation type and confound the observed relationships between plant and microbial diversity. For example, Louisson et al.,^16^ observed significant legacy effects on soil microbial community composition and functional potential more than 10 years following land use conversion from grassland to forest and more than 5 years following land use conversion from forest to grassland. These factors highlight the complexities of understanding plant-microbe interactions.

Reforestation is an important tool for ecological restoration. It involves planting areas previously cleared of forest^17^ in attempts to reverse or at least slow rates of global ecosystem degradation.^18^ However, the success of restoration projects is impacted by factors including plant density, composition, and diversity.^19^ For example, mixed species plantations can support increased tree growth^20^ and higher arthropod species diversity,^21^ relative to monoculture stands. Restoration plantings can impact ecosystem functioning,^22^ with more diverse and complex environmental plantings resulting in higher carbon soil stocks relative to monoculture.^23^ Reduced plant diversity has also been linked to reduced soil respiration rates, which can affect nutrient cycling.^24^ However, most studies examining the microbial effects of reduced plant diversity or monoculture systems focus on their taxonomic distributions. It is particularly important to understand the functional response of microbial communities in restoration projects, as soil microbial communities play an essential role in soil biogeochemical cycling.^25^^,^^26^ Therefore, monitoring soil microbial community dynamics and functional potential exposed to different forest restoration strategies can provide a holistic overview of their impact on ecosystem functioning and biogeochemical cycling.

**Figure 1:**
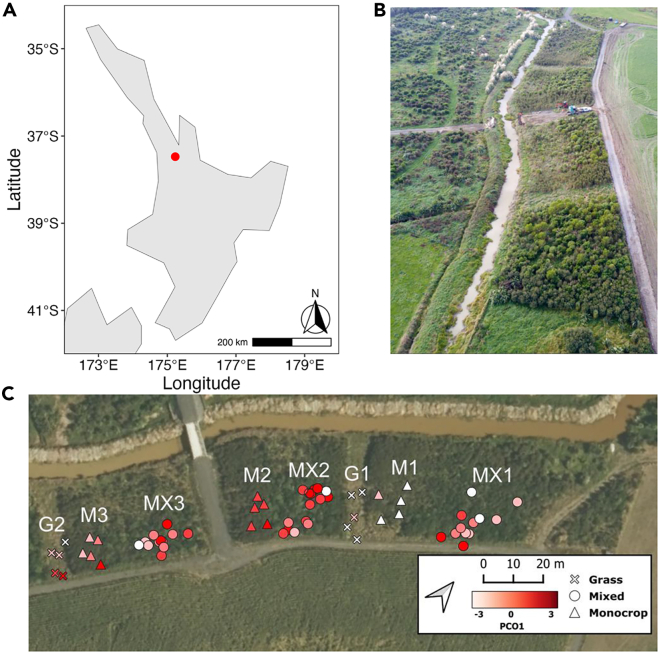
Site information (A) Location of the experimental site (Lat: −37.47389, Long: 175.23194) in the context of northern New Zealand and (B) aerial imagery of the plots. (C) Location of each sample location from within their respective subplots. MX represents a mixed plot, M represents a monoculture mānuka plot and G represents a grass plot. The points are colored based on the scores of axis 1 of a PCO analysis of a Euclidean distance matrix of the chemical profiles of each sample (see Figure S1). The PCO axis 1 explained 31.8% of the variation in the composition of the chemical profiles which significantly differed between plots (PERMANOVA R^2^ = 39, *p* < 0.001).

Most studies that have investigated the effects of monocultural stands on soil microbial communities have focused on food crops,^27^^,^^28^^,^^29^ which are typically grown in heavily managed environments, due to their economic importance. Since monocultural tree plantations are also managed systems, they can offer insights into microbial communities as compared to within unmanaged environments, and potentially inform ecological restoration decisions. Our study compares microbial communities and their functional potential in soils beneath stands of both monocultural mānuka (*Leptospermum scoparium* J.R. Forst. & G. Forst.) and mixed native woody species (Table S1) from an experimental restoration site established on converted dairy pasture (Figure 1).^30^ Using 16S and ITS rRNA gene amplicon sequencing alongside shotgun metagenomic sequencing, we examined the effects of reduced plant diversity on the soil bacterial and fungal communities and their functional potential. We investigate the following questions: (1) Does reduced plant diversity translate to reduced taxonomic diversity and functional potential of soil microbial communities under mānuka trees? (2) Do soil bacterial and fungal communities under mānuka trees differ in their spatial patterns? (3) Do the dominant mechanisms structuring these bacterial and fungal communities differ? (4) Are specific functional pathways impacted by the reduced diversity of neighboring woody plant? We provide insights into how aboveground restoration practices influence soil microbial communities and the implications of reduced plant diversity for ecosystem productivity, which can inform conservation decisions.

## Results

### Taxonomic and functional diversities across the site

**Figure 2:**
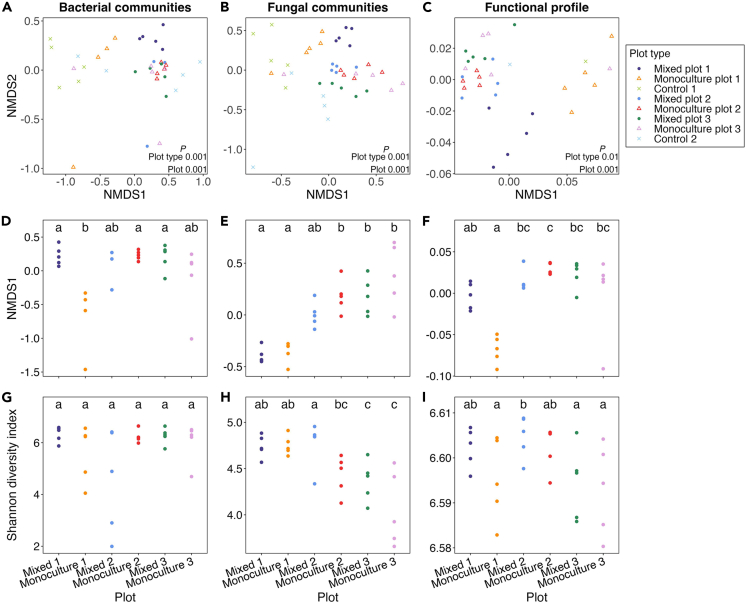
Taxonomic and functional diversities across the site Bray–Curtis dissimilarity based non-metric multidimensional scaling (nMDS) ordinations of (A) bacterial, (B) fungal community composition (based on ASVs) and (C) functional potential (based on SEED subsystem level 4 categories). P-values from PERMANOVAs assessing site-type effects are displayed in the bottom right corner of each plot. (D–F) Axis 1 scores of Bray–Curtis based nMDS ordinations of (D) bacterial, (E) fungal community composition (based on relative abundances of ASVs) and F functional potential (based on SEED subsystem level 4 categories). (G–I) Shannon diversity index values of the G bacterial communities, (H) fungal communities and (I) functional profiles. Plots with different letters within each panel indicate significant differences from each other (Dunn’s *p* < 0.05). Shannon diversity index values were quantified using the rarefied data.

After quality filtering the amplicon data, we obtained 2,088,877 bacterial and 2,995,189 fungal sequencing reads. For the shotgun metagenomic data, an average of 17.8 million sequences per sample remained after quality filtering. When the data were grouped by plot type (grass, monoculture, and mixed plots), alpha diversity (calculated from rarefied amplicon sequence data) did not significantly differ among the plot types for either bacterial or fungal communities (Shannon diversity index, Dunn’s *p* > 0.05, Figures S2A and S2B). Similarly, the alpha diversity of the functional data were comparable between the monoculture and mixed plots (Shannon diversity index, Kruskal Wallace *p* > 0.05, Figure S2C). For the functional data, we only had two grass samples (one representative sample for each control plot). Therefore, we did not compare the alpha diversity for the grass plot type. We also compared alpha diversity among the different planting treatments (the six individual monoculture and mixed plots, excluding the grass plots, Figure 2). For the bacterial communities, alpha diversity also did not significantly differ among each of the six treatment plots (Shannon diversity index, Dunn’s *p* > 0.05, Figure 2G). However, for the fungal communities, we observed a general decrease in alpha diversity, with Plot One having higher diversity, Plot Two intermediate and lowest in Plot Three (Shannon diversity index, Dunn’s *p* > 0.05 Figure 2H). The alpha diversity of the functional data in mixed Plot Two significantly differed from the Monoculture Plot One and Monoculture and Mixed Plot Three (Shannon diversity index, Dunn’s *p* < 0.05, Figure 2I).

### Microbial taxonomic and functional potential differed among plot types and individual plots

We identified 20,113 unique bacterial ASVs from the CSS normalized data, representing 33 phyla, 291 families and 622 genera. Of the top ten most abundant bacteria phyla among the plot types (monoculture, mixed and grass), the phylum *Firmicutes* was the most different in relative abundance between the planted plots (monoculture and mixed) and the grass plots, with a relative abundance of 15.6% in the monoculture, 13.5% in the mixed and 5% in the grass plots (Figure S3A). For the fungal communities, 5160 ASVs were identified and were classified into 16 phyla, 321 families and 643 genera. Of the top ten most abundant fungal phyla, the phyla *Ascomycota* and *Basidiomycota* were the most different in relative abundance among the plots, with relative abundances of 49.7% and 34.2% in the monoculture plots, 58.5% and 26.6% in the mixed plots and 63.8% and 19.4% in the grass plots respectively (Figure S3B). For the shotgun metagenomic reads that could be annotated in MEGAN, 3,248,117 reads were classified into 818 SEED subsystems, while 351,186,080 reads were classified into 12,701 KEGG Orthology groups. At SEED and KEGG Level 1 categories, no functional groups significantly differed in relative abundance (Mann Whitney U, *p* > 0.05, Figure S4). Procrustes rotation analyses indicated a significant correlation between the community composition of the bacteria and fungi (*r* = 0.85, *p* < 0.001), and between the bacterial communities (ASVs) and the functional profiles (SEED Level 4) (*r* = 0.80, *p* < 0.01). The bacterial and fungal taxonomic composition and the functional potential of the soil microbial communities were significantly different among plot types (monoculture, mixed, and grass plots) and among the eight plots (PERMANOVA *p* < 0.01; Figures 2A–2C; Table 1). Although relative abundances of the top ten most abundant phyla varied little, pairwise PERMANOVA indicated that bacterial and fungal community compositions significantly differed among the three plot types (monoculture, mixed and grass) at the ASV level (PERMANOVA *p* < 0.05; Table S4). For both bacterial and fungal communities, <1% of the variation between the composition of the communities in the monoculture and mixed plots was attributed to plot type, while >10% of the variation between the communities in both planted plot types (monoculture and mixed) and the grass plots could be attributed to plot type, based on the R^2^ values (Table S4). Individual plots (the eight plots) explained the greatest amount of compositional variation for the bacterial (R^2^ = 25%), fungal (R^2^ = 23%) and functional (R^2^ = 28%) data (Table 1). Plot type (grass, monoculture and mixed plots) explained a further 16%, 13% and 9% of the variation in the composition of the bacterial and fungal communities and the functional profile, respectively (Table 1).

**Table 1:** PERMANOVA results of bacterial and fungal community composition and functional potential

	Source of variation	d.f.	Sums of Square	_R_2	F	*P*
Bacterial communities	Plot type	2	1.30	0.16	4.00	**< 0.001**
	Plot	5	2.05	0.25	2.51	**< 0.001**
	Residuals	29	4.72	0.58		
Fungal communities	Plot type	2	1.25	0.13	3.40	**< 0.001**
	Plot	5	2.16	0.23	2.35	**< 0.001**
	Residuals	32	5.88	0.63		
Functional profile	Plot type	2	0.02	0.09	1.67	**< 0.01**
	Plot	5	0.06	0.28	2.12	**< 0.001**
	Residuals	24	0.15	0.63		
Soil chemistry profile	Plot type	2	60.11	0.17	6.28	**< 0.001**
	376 Plot	5	137.84	0.39	5.76	**< 0.001**
	Residuals	32	153.05	0.44		

Table indicates the partitioning of variation and tests for plot type and individual plots (1–8). R2 is the square root of the component of variation attributable to that factor in the model, in units of Bray-Curtis distance. D.f.: degrees of freedom.

We extracted the first axis data scores of each nMDS ordination to examine the difference in microbial composition between the individual treatment plots (monoculture and mixed plots). For the bacterial data, plots did not differ from each other (Dunn’s *p* > 0.05), excluding the Monoculture Plot One, which significantly differed (Dunn’s *p* < 0.05) from all other plots except Monoculture Plot Three (Figure 2D). For the fungal data, both Monoculture and Mixed Plots One significantly differed (Dunn’s *p* < 0.05) in composition from Monoculture and Mixed Plots Three, while data from Monoculture and Mixed Plots Two were intermediate; Mixed Plot Two was comparable to both Plots One and Three, while Monoculture Plot Two was comparable to both Three Plots (Figure 2E). A comparable trend to that observed in the bacterial data, was seen in the functional data (Figure 2F).

### Influence of soil chemistry factors

Procrustes rotation analyses indicated a strong, significant correlation between the ordination of the soil chemistry data and each of the bacterial (*r* = 0.74, *p* < 0.001), fungal (*r* = 0.71, *p* < 0.001), and functional (*r* = 0.75, *p* < 0.001) composition dataset ordinations. Individual plots explained the greatest amount of variation in the soil chemical profiles (R^2^ = 39%), while plot type explained a further 17% (PERMANOVA *p* < 0.001, Table 1). The composition of the bacterial and fungal communities and the functional profiles sampled under mānuka trees, in both plot types (monoculture and mixed), were each significantly related to pH and water content, while the composition of the fungal communities was also related to exchangeable potassium (Table S5). For the communities' taxonomic composition and functional profile, pH and water content explained the greatest proportional variability. pH explained 21%, 10% and 19% of the variability for the bacterial, fungal, and functional data, respectively, while water content explained 10%, 12%, and 7%, respectively (Table S5).

### Spatial and environmental drivers of microbial communities

Variance partitioning of the beta diversity into environmental and spatial components for the bacterial communities showed that soil chemistry factors explained the largest proportion of variation (11%), while spatial features explained 6% of the variation and a further 16% of shared variation (Table S6). For the fungal communities, the variance explained solely by soil chemistry and spatial components was similar, explaining 6% and 5%, respectively and they explained a further 18% of shared variation (Table S6). For the functional data, soil chemistry accounted for 5% of the variance, while spatial components explained 4%, and a further 18% of shared variation (Table S6).

**Figure 3:**
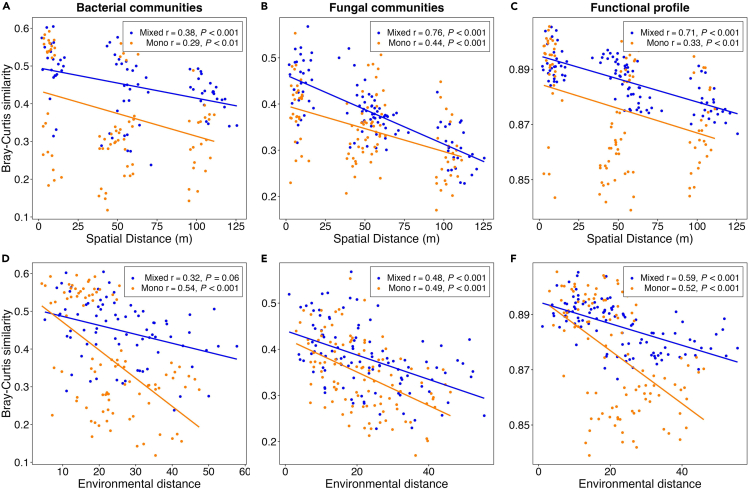
Spatial and environmental drivers of microbial communities Distance-decay relationship of Bray-Curtis similarity and (A–C) geographic distance (m) (based on Euclidean distance of coordinates) and (D–F) environmental distance (based on Euclidean distance of soil chemistry variables). Results of the Mantel tests are displayed in the plots.

Distance-decay plots showed that the pairwise composition of bacterial and fungal communities and the functional potential of the soil communities in the monoculture and mixed plots became consistently more different with increasing geographic distance between samples (Figures 3A–3C, Mantel *p* < 0.01). For the taxonomic profiles, the strength of the geographic distance-decay relationships (based on Mantel correlation coefficients (*r*) as an effect size measure) was stronger in the fungal communities relative to the bacterial communities for both plot types (Pearson’s r; mixed plots *r* = 0.38 and *r* = 0.76, monoculture plots *r* = 0.29 and *r* = 0.44 for bacterial and fungal communities, respectively). A similar relationship between the taxonomic composition of the communities (bacterial and fungal) and the functional potential of the communities was observed for environmental distance, whereby the samples became more different with increasing differences in soil chemical conditions (Figures 3D–3F). For the bacterial communities, the environmental distance-decay relationship was stronger in the monoculture plots (*r* = 0.54), relative to the mixed plots (*r* = 0.32). However, the strength of the relationship was comparable between the plot types for the fungal data (*r* = 0.49, *r* = 0.48 for the monoculture and mixed plots, respectively).

### Distinct community assembly processes in bacterial and fungal communities

In the monoculture and mixed plots, we identified the dominant community assembly processes for the bacterial and fungal communities. We found homogeneous selection accounted for 98.7% of assembly in the bacterial communities. For the fungal communities, homogeneous selection accounted for 46.9%, while dispersal limitation accounted for 36.1% of fungal community assembly (Table 2).

**Table 2:** Community assembly processes

	Homogeneous selection (%)	Heterogeneous selection (%)	Dispersal limitation (%)	Homogenising dispersal (%)	Undominated (%)
Bacterial communities	98.67	0	0.33	0.33	0.67
Fungal communities	46.90	0	36.09	0	17.01

Percent contribution of various ecological processes, determined as the primary assembly process governing the turnover of bacterial and fungal communities, for each pairwise comparison between each soil community residing under mānuka trees.

### The resemblance of functional profiles among communities under different tree species

**Figure 4:**
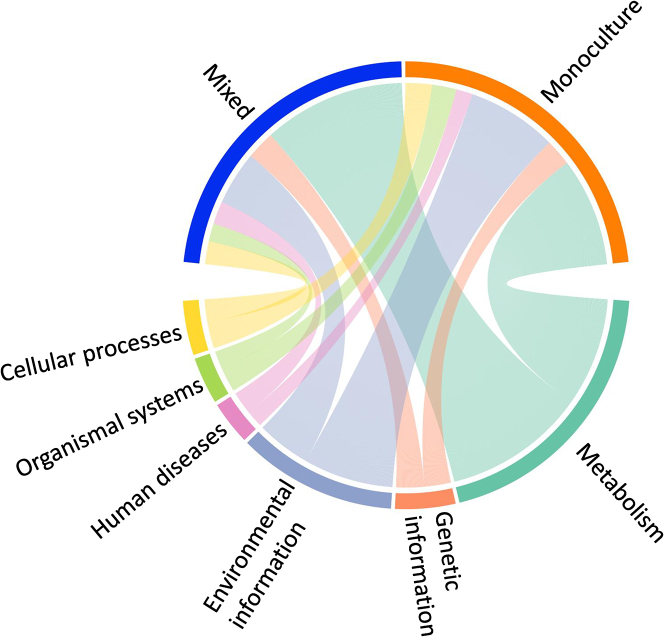
The resemblance of functional profiles among communities under different tree species Chord diagram of the identified indicator functional groups (based on Level 4 KEGG categories) for the soil communities under the mānuka trees in the mixed and monoculture plots and their distribution across the Level 1 KEGG categories. The width of each connecting arc represents the proportion of Level 4 indicator functional groups identified in the mixed and monoculture plots that are classified by each Level 1 category (for example Cellular processes or Metabolism).

For the KEGG functional data, the largest percentage of reads were classified within the ‘metabolism’ functional group, making up an average of 44.5%. The second-highest category was ‘environmental information processing’, which accounted for 22.5% of all reads (Figure S4A). However, at the lower functional levels, we did observe differences in functional groups between the plot types (monoculture and mixed). An indicator species analysis (IndVal) identified KEGG functional categories (Level 4) that were significantly associated with the monoculture and mixed plots in the communities residing under the mānuka trees. There were 598 functional groups that were identified as representative or ‘indicator functional groups’ of the monoculture plots, while 593 were associated with the mixed plots. For both plot types, when grouped by the Level 1 category, the indicator functional groups were mainly associated with the metabolism and environmental information processing categories. For the monoculture plot data, 37.1% of the indicator functions were from the metabolism category, while 30.8% were from the environmental information processing category. For the mixed plot data, a larger proportion was categorized as metabolism functional groups (49.4%), while 18.7% were from the environmental information processing category (Figure 4).

### Higher abundance of metabolism functional gene groups in mixed plots

**Figure 5:**
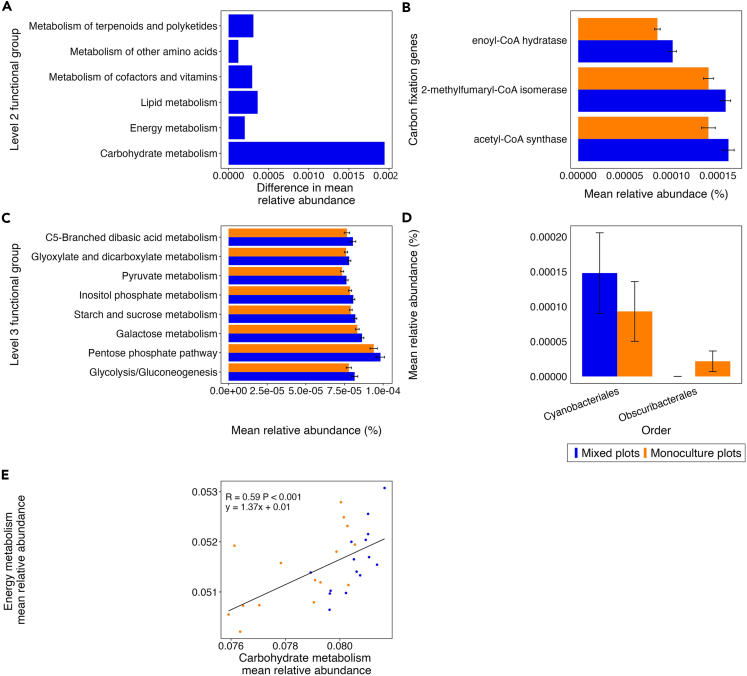
Higher abundance of metabolism functional gene groups in mixed plots (A) Difference in relative abundance of the Level 2 KEGG categories, within the Level 1 category metabolism that significantly differed in relative abundance between the monoculture and mixed plots (Mann-Whitney U *p* < 0.05). (B and C) Mean relative abundance of the (B) Level 4 KEGG categories, within the Level 3 prokaryotic carbon fixation category and (C) Level 3 KEGG categories, within the Level 2 category carbohydrate metabolism, which significantly differed (Mann-Whitney U *p* < 0.05) in relative abundance between the monoculture and mixed plots. (E) The relationship between the mean relative abundance of carbohydrate metabolism and energy metabolism in the monoculture and mixed plots. Pearson’s correlation was used to measure the strength of the relationships. (D) The mean relative abundance of orders within the phylum *Cyanobacteria* in the monoculture and mixed plots. Error bars represent the standard error.

Given soil microbial communities' vital role in cycling nutrients, we analyzed whether metabolism pathways differ between soils in monoculture and mixed plots. Six metabolism Level 2 categories significantly differed in relative abundance between the monoculture and mixed plots (Figure 5A, Mann-Whitney U *p* < 0.05). All six Level 2 metabolism-related pathways were more abundant in the mixed plots. The carbohydrate metabolism category differed most in mean relative abundance between the plot types (Figure 5A). We also compared whether metabolism pathways differed among communities under mānuka trees in the mixed plots and the seven other tree species. There were no significantly different Level 2 metabolism-related pathways when data were averaged among the seven tree species and when compared separately for each tree species (Mann-Whitney U *p* > 0.05).

Within the carbohydrate metabolism category, eight Level 3 pathways significantly differed in relative abundance between the soil communities under mānuka trees in the monoculture and mixed plots (Figure 5C, Mann-Whitney U *p* > 0.05). All eight functional groups were more abundant in the mixed plots (Figure 5C). A moderately strong relationship existed between the relative abundance of carbohydrate and energy metabolism pathways (Figure 5E, slope = 1.37, *R* = 0.59, *p* < 0.001). Therefore, we further investigated the Level 4 KEGG categories of energy metabolism. Sixty-two energy metabolism cycling genes significantly differed in relative abundance between the monoculture and mixed plots (Figure S5, Mann-Whitney U *p* < 0.05). Thirty-six of these functional gene groups were more abundant in the mixed plots, while twenty-six were more abundant in the monoculture plots (Figure S5). Of the 62 energy metabolism cycling genes, all three genes from the Level 3 category prokaryotic carbon fixation that significantly differed in relative abundance between the plot types were more abundant in the mixed plots (Figure 5B). In the taxonomic data, the photosynthetic taxa Cyanobacteria were more abundant in the mixed plots (Figure 5D).

## Discussion

We confirmed that the underlying mechanisms structuring bacterial and fungal communities differed, likely impacting how each taxonomic group responds to, and affects, restoration projects. Importantly, we highlight the impact monocultural stands may have on reducing certain energy metabolism pathways. Overall, this study indicates that examining the microbial components of ecosystems can be beneficial for understanding processes occurring during ecological restoration.

### Aboveground diversity has a stronger influence on community composition than richness

In agreement with Prober et al.,^5^ and Wardle,^9^ we show that reduced plant diversity did not consistently influence the alpha diversity of soil bacterial and fungal communities or the alpha diversity of the functional potential of these communities. However, our results contrast with other studies, which showed positive relationships between plant diversity and microbial alpha diversity.^31^^,^^32^^,^^33^ One such study comparing the recovery of soil biodiversity in degraded land observed that afforestation with mixed native tree species led to the recovery of microbial diversity and increased functional characteristics (for example heightened microbial enzyme activity), while monocultural plantations of exotic forests did not.^32^ An explanation for the contrasting results could be the difference in time since restoration planting. Wu et al.,^32^ examined the effects on microbial communities sixty years after forests were established, while our study was on plantings that were still relatively young; sites were sampled just five years after trees were planted. As microbial diversity can take years to recover from land degradation alongside time since restoration,^33^ we may see greater differences in taxonomic and functional alpha diversity as time goes on. This study has established the baseline for future monitoring.

In contrast to our results on alpha diversity, plot type (monoculture, mixed and grass plots) did influence patterns in community composition and functional potential. We observed significantly different bacterial and fungal community composition among the plot types. However, this was principally due to differences between the planted plots (monoculture and mixed) and the grass plots. The difference in bacterial community composition between the planted and grass plots was largely driven by the reduced relative abundance of the phylum *Firmicutes* in the grass plots. This is surprising as *Firmicutes* is often associated with pasture and livestock.^34^ Therefore, we would have expected a higher abundance in the grass plots. However, *Firmicutes* has been previously identified as a dominant phylum associated with mānuka plants, particularly in mānuka populations located in the Waikato region of New Zealand,^35^ where our study site was located. Additionally, the difference in fungal community composition between the planted and grass plots was partly due to a higher relative abundance of the phylum *Basidiomycota* in the mixed plots and further increases in the monoculture plots. Records of fungi that closely associate with *Leptospermum* (the genus of mānuka) indicate that, of the 413 taxa identified, over half of these species were from the *Basidiomycota* group.^36^ The observed heightened relative abundance of microbes previously identified as closely associated with mānuka suggests that the host plant does have a role in shaping the composition of microbial communities, supporting the findings of another study where a consistent core microbiome in the phyllosphere of mānuka was determined.^35^

Although the composition of the bacterial and fungal communities was statistically different between the monoculture and mixed plots, very little variation (<1% for both taxonomic groups) was attributed to the difference in tree diversity. Our results are consistent with a study in Wales examining the effect of native forest complexity on microbial communities.^37^ They showed microbial communities in tree-planted plots differed from neighboring agricultural soils, while monoculture plots and those planted with mixtures of three species hosted similar microbial communities.^37^ We may not have observed clear differences in alpha diversity and limited compositional differences between the plot treatments because mānuka is a native pioneer species, and mānuka-dominated shrublands are widespread throughout New Zealand.^38^ Native trees may be adapted to local conditions, and the resident soil microbes are likely to have co-evolved with these native plants and the compounds they exude.^39^ As soil microbial biomass recovers at a faster rate under native trees, relative to exotic species,^40^ the limited differences in diversity observed in our study may be partly attributed to mānuka supporting a diverse group of local microbes due to a co-evolved relationship. Our results suggest that some form of tree cover, whether monocultural or mixed plantings, influences soil microbial community composition, and these changes can be observed within five years of restoration.

### Fungal communities exhibit stronger compositional differences than bacterial communities

More of the bacterial and fungal community composition variation was explained by the individual plots, rather than plot type (monoculture, mixed or grass). As the ordinations of the taxonomic community compositions were correlated with the ordinations of the soil chemistry profiles, the difference among the plots in the community composition are likely influenced by the differences in the soil environments. For example, regarding the bacterial data, Monoculture Plot One had the most distinct community composition relative to the other plots. This likely reflects the soil chemical conditions as Monocrop Plot One also differed most in soil chemical composition. Additionally, some of the mānuka plants in this plot showed signs of chlorosis, possibly due to the higher concentrations of nutrients.^30^ Although we did observe differences in microbial composition between planting treatments, our results further support that differences in edaphic traits are the dominant factors influencing community turnover^41^^,^^42^^,^^43^ and that legacy effects from historical land use can be a stronger determinant of community structure than existing vegetation.^16^^,^^44^ In line with other studies,^15^^,^^45^^,^^46^ the alpha diversity and community composition of the fungal communities exhibited more variation among individual plots compared to the bacterial communities. Due to bacterial growth rates and their unicellular nature, they are often considered more robust to disturbance or environmental changes relative to fungi.^47^ Additionally, when the effects of soil moisture on microbial communities were analyzed at non-extreme ranges, fungal communities were identified as more sensitive indicators of soil moisture.^15^ Therefore, the different patterns among the taxonomic groups in our data could be due to the higher sensitivity of fungal taxa to the prevailing environmental conditions. However, according to our variance partitioning results, soil chemical variables explained more variation than spatial components in bacterial community composition. In contrast, similar levels of variance were explained by each category for the fungal community composition. Therefore, the different patterns observed between the bacterial and fungal communities could be due to the difference in relative importance that environmental and spatial factors have on influencing community composition.

### Distinct mechanisms regulate bacterial and fungal community assembly

Different factors regulate the relative influences of environmental and spatial components on the structure of bacterial and fungal communities. Still, our study indicates that the different dispersal capacities of the taxonomic groups largely regulate this balance. We showed that bacterial and fungal communities were regulated by homogeneous selection; however, while this was the dominant assembly process for the bacterial communities, fungal communities were also heavily structured by dispersal limitation. This is in line with some previous studies which showed that fungi were more dispersal limited than bacteria.^47^^,^^48^^,^^49^ However, our study showed that this pattern is maintained even at a relatively small scale (single site). These results support the hypothesis that larger organisms have reduced dispersal.^49^ As bacteria and fungi differ in growth traits, the fungal communities are likely more limited by dispersal as they are typically larger-celled organisms than bacteria.^47^ The influence of dispersal limitation on the fungal communities likely explains why we see more distinct differences in community composition of the fungal communities and a stronger geographic distance-decay pattern compared to the bacterial communities. Additionally, fungi commonly display filamentous growth and form close associations with plant roots.^50^ Therefore, this tight association of fungi with plant roots may increase the influence of dispersal limitation in the fungal communities. Dispersal limitation in fungal communities increased over time in a reforestation project, which could be attributed to increased root development and, in turn, increased associations between plant and soil fungal communities.^51^ Therefore, we may see the influence of dispersal limitation and stochastic processes in structuring fungal communities increase over time in our site.

The bacterial communities were predominantly regulated by homogeneous selection, consistent with the stronger influence of soil chemical variables relative to spatial factors on bacterial community composition in the variance partitioning. Homogeneous selection reduces stochasticity and leads to higher compositional similarities between communities.^52^ Therefore, the dominant role of selection in structuring bacterial communities likely explains the higher similarity in community composition among the individual plots relative to the fungal communities. Bacterial communities often exhibit wider niche breadth and are more metabolically flexible than fungal communities.^53^ Although soil water and pH explained the largest proportion of variation for both bacterial and fungal community composition, we still observed lower variation in composition among the sites in the bacterial communities. Therefore, higher adaptability to more diverse environments, combined with the influence of dispersal limitation on fungal communities, may explain why we observed consistently lower phylogenetic turnover in the bacterial communities.

These results suggest that bacterial communities may recover faster than fungal communities, post-restoration. However, the influence of dispersal limitation on structuring bacterial communities can increase with spatial scale.^54^ Therefore, the scale of the project will be relevant to the interpretation of results and management of the restoration project. Although it is often presumed that microbial communities independently recolonize areas after reforestation, dispersal limitation can be a limiting factor in the recolonization of restored ecosystems.^51^^,^^55^ As microbial communities can take years to respond to land restoration, rewilding of microbial communities has been suggested to mitigate the constraints of dispersal limitation through methods such as inoculation.^55^ Therefore, defining the mechanisms structuring microbial communities could assist with determining target communities and potentially improve and/or speed up restoration success.

### Reductions in plant diversity can impact metabolic processes

The differences in the microbial community composition were reflected in the functional potential of the soil communities, with more of the variation in the functional composition explained by individual plots compared to monoculture versus mixed plantation comparisons. Additionally, we observed relatively consistent abundances of functional groups at the high subsystems (Level 1), suggesting that the dominance of homogenizing selection and reduced phylogenetic turnover in the bacterial communities has a role in homogenizing the functional profiles across the site. Nevertheless, the planting treatment (monoculture or mixed) did have a role in shaping the functional profiles of the communities as we identified functional genes highly representative of each plot type. The representative genes identified in the mixed plots were largely related to metabolism. Overall, the functional analyses suggest that increased plant diversity may increase the overall productivity of soil environments, in agreement with other studies.^8^

Different plant species vary in the production of chemical compounds and release of exudates, including sugars and secondary metabolites,^56^ and increased plant diversity has been shown to enhance the quantities or diversity of rhizodeposits and dissolved organic matter.^57^ Therefore, the higher mean relative abundance of carbohydrate metabolism and functional pathways related to the metabolism of starch and sugars observed in the mixed plots could reflect higher diversity of substrates available in the soil environment. This is in agreement with other studies which showed increased carbohydrate metabolism after conversions of monoculture forests to mixed plantations^58^ and increased litter leaf diversity,^59^ suggesting that increased plant diversity does directly influence the metabolism activity of soil microbial communities.

The compounds in the root exudates, such as sugars and amino acids, are directly assimilated by soil microbes and influence community dynamics;^60^ higher inputs can increase rates of microbial activity.^61^ Our study supports this, as more genes relating to energy metabolism that significantly differed between the monoculture and mixed plots had higher relative abundance in the mixed plots. As we observed a positive relationship between carbohydrate metabolism and energy metabolism, the heightened number of energy metabolism gene groups that were more abundant in the mixed plots could be due to an enhanced diversity of exudates offered by increased plant diversity.^56^ Within the energy metabolism group, the three prokaryotic carbon fixation genes that significantly differed in relative abundance between the plot types exhibited higher relative abundance in the mixed plots, alongside the heightened relative abundance of carbon-fixing cyanobacteria. Soil organic carbon and root exudates have been identified as factors that promote the carbon fixation activities and composition of soil microbial communities.^62^ Therefore, our results suggest that reduced plant diversity could influence important processes, such as microbial carbon fixation rates, which are thought to be important contributors to soil carbon sequestration rates.^63^ However, measuring rates of these processes *in situ*, such as carbon fluxes or exudate composition, would strengthen our deductions.

### Conclusions

Our study demonstrates that although soil environmental conditions and geographic distance were more important determinants of microbial community dynamics than the diversity of neighboring trees species, reduced plant diversity also influenced the composition and functioning of soil microbial communities. Our site is still relatively new, so we expect the differences between the monocultural and mixed plots to increase over time.^7^ Therefore, tree species selection during restoration planning can have important implications for ecosystem recovery. Our findings indicate that fungal communities may be more limited by dispersal than bacterial communities, suggesting fungal communities may take longer to respond to restoration efforts due to lower rates of passive colonization. Increasing our understanding of the mechanisms structuring microbial communities can assist in monitoring and evaluating restoration success.

### Limitations of the study

The DNA-based amplicon sequencing and shotgun metagenomics methods used in this study fail to discern between active and inactive community members or genes, which can result in an overestimation of microbial diversity.^64^^,^^65^ Additionally, shotgun metagenomic sequencing is not a reflection of a community’s metabolic activity, but rather a representation of the functional potential of a community.^66^ Therefore, the differences in relative abundance among functional groups observed in the planting treatments do not necessarily indicate differences in expression of certain genes. Using RNA-based methods to target the active community could offer a more accurate representation of the diversity and functioning of the soil microbial communities.

## STAR★Methods

### Key resources table

**Table:** 

REAGENT or RESOURCE	SOURCE	IDENTIFIER
**Deposited data**		

Raw sequence data	This paper, National Center for Biotechnology Information (NCBI)Sequence Read Archive (SRA)	SRA: PRJNA1046246

**Oligonucleotides**		

341F	Klindworth et al.,^70^	5′-TCGTCGGCAGCGTCAGATGTGTATAA GAGACAGCCTACGGGNGGCWGCAG-3′
785R	Klindworth et al.,^70^	5′-GTCTCGTGGGCTCGGAGATGTGTATA AGAGACAGGACTACHVGGGTATCTAATC C-3′
fITS7	Ihrmark et al.,^71^	5′ TCGTCGGCAGCGTCAGATGTGTATAAG AGACAGGTGARTCATCGAATCTTTG 3′
ITS4	Ihrmark et al., 2012	5′ GTCTCGTGGGCTCGGAGATGTGTATAA GAGACAGTCCTCCGCTTATTGATATGC 3′

**Software and algorithms**		

R version 4.3.0	R Core Team^72^	https://cran.r-project.org
Primer version 7	Clarke and Gorley^73^	PRIMER-e » Primer Version 7
MAFFT (v7.505)	Katoh and Standley^81^	MAFFT - a multiple sequence alignment program (cbrc.jp)
trimA1 (v 1.2 rev 59)	Capella-Gutiérrez et al.,^82^	trimal [trimAl] (cgenomics.org)
FastTree (v 2.1.10)	Price et al.,^83^	FastTree 2.1: Approximately-Maximum-Likelihood Trees for Large Alignments (microbesonline.org)
FastQC (v0.11.7)	Andrews,^84^	Babraham Bioinformatics - FastQC A Quality Control tool for High Throughput Sequence Data
Trimmomatic (v0.38)	Bolger et al.,^85^	USADELLAB.org - Trimmomatic: A flexible read trimming tool for Illumina NGS data
Prodigal (v.2.6.3)	Hyatt et al.,^86^	GitHub - hyattpd/Prodigal: Prodigal Gene Prediction Software
DIAMOND (v2.1.6)	Buchfink et al.,^87^	GitHub - bbuchfink/diamond: Accelerated BLAST compatible local sequence aligner.
MEGAN6	Huson et al.,^88^	MEGAN6 | Universität Tübingen (uni-tuebingen.de)

### Resource availability

#### Lead contact

Further information and requests for resources should be directed to and will be fulfilled by the lead contact, Ziva Louisson (zlou499@aucklanduni.ac.nz).

#### Materials availability

This study did not generate new unique reagents.

#### Data and code availability

The amplicon and shotgun metagenomic sequence data have been deposited at NCBI Sequence Read Archive and are publicly available as of the date of publication. Accession numbers are listed in the key resources table.

This paper does not report original code.

Any additional information required to reanalyse the data reported in this paper is available from the lead contact upon request.

### Method details

#### Site description and sample collection

An experimental site was established on 1.6 ha of the riparian zone of Lake Waikare in the Lower Waikato region of New Zealand (Figure 1).^30^ We used the R package ‘rnaturalearth’ to map the site location.^67^ The soil type at the experimental site was classified as Ultic, using the New Zealand Soil Classification (equivalent to Acrisol in the World Reference Base, IUSS, 2015).^68^ Before the site was established, the land was dairy pasture until 2017, when it was converted into the experimental setup (planting occurred in June and October 2017). The experimental site is a 272 m × 30 m riparian strip planted at a density of ∼1 plant/m2. The site consists of ten subplots; three unplanted grass plots, three monoculture plots planted with a local variety of *Leptospermum scoparium* known as swamp mānuka (henceforth, mānuka) and four planted with a mixture of twenty species native to New Zealand (Table S1), including mānuka, which represents the natural flora of riparian or wetland ecosystems in the Lower Waikato region. Mānuka was selected for the monoculture plantations due to its role as a pioneer plant which often establishes in disturbed environments. It commonly grows in quasi-monoculture until later successional species emerge.^69^

For this study, eight of the planted subplots were selected to provide an equal number of mānuka monoculture and mixed species plots, plus two grass control plots. Plots were sampled in January 2022. For microbial analyses, we collected five soil cores (0-10 cm depth, 2.5 cm diameter) 1 m from the base of the select plant, in a radial pattern. Five mānuka plants were selected from each of the monoculture and mixed species plots (n = 30). An additional eight tree species were chosen, representing species common within each mixed plot, with one of each species sampled per mixed species plot (n = 24; Table S1). Additionally, five soil samples (0-10 cm depth, 2.5 cm diameter) were taken from each grass control plot (n = 10). All samples were kept on ice until they were transferred to -20°C storage until DNA extraction. We composited an additional five soil cores per tree (n = 54) and an extra five individual cores per grass plot (n = 10) for soil chemical analyses (Table S2).

#### DNA extraction, PCR and sequencing

Each sample was thawed, manually homogenised and pooled to form a composite sample per tree. We used DNeasy PowerSoil Pro kits (Qiagen, Valencia, CA, USA) to extract genomic DNA from 250 mg of each soil sample according to the manufacturer’s instructions, with the following modifications: (i) a Qiagen TissueLyer II instrument (Retch) was used for mechanical lysis for 4 min at a frequency of 30 Hz; and (ii) after adding the elution buffer, the plates were incubated at room temperature for 5 min. The extracted DNA (n = 64) was placed in -20°C storage until required. To characterise the bacterial and fungal taxonomic diversity and composition of each sample, we amplified the V3/V4 region of 16S ribosomal RNA genes through PCR using the primers 341F (5′-TCGTCGGCAGCGTCAGATGTGTATAAGAGACAGCCTACGGGNGGCWGCAG-3′) and 785R (5′-GTCTCGTGGGCTCGGAGATGTGTATAAGAGACAGGACTACHVGGGTATCTAATC C-3′).^70^ We amplified ITS2 genes using the primers fITS7 (5′ TCGTCGGCAGCGTCAGATGTGTATAAGAGACAGGTGARTCATCGAATCTTTG 3′) and ITS4 (5′ GTCTCGTGGGCTCGGAGATGTGTATAAGAGACAGTCCTCCGCTTATTGATATGC 3′).^71^ PCR primers were modified with Illumina DNA sequencing adapters (underlined), optimised for use on Illumina MiSeq platforms. We amplified bacterial DNA from each sample using the following conditions: (i) 95°C for 3 min; (ii) 25 cycles of 95°C for 30 s, 55°C for 30 s, and 72°C for 30 s; and then (iii) 72°C for 5 min. Fungal DNA was amplified using these conditions: (i) 94°C for 5 min; (ii) 30 cycles of 94°C for 30 s, 52°C for 30 s, and 72°C for 45 s; and then (iii) 72°C for 10 min. As per the manufacturer's instructions, we purified the PCR products using DNA clean-up kits (Zymo Research). We quantified the concentrations of the purified PCR products using a Qubit double-stranded DNA HS assay kit (Thermo Fisher Scientific) and normalised samples to a standard concentration (4 ng/μL). The purified products were submitted to New Zealand Genomics Ltd. (Auckland, New Zealand), barcoded using Nextera XT dual indices, pooled, and sequenced on an Illumina MiSeq instrument using V3 chemistry, producing 2 x 300 bp paired-end reads. To characterise the functional potential of the soil microbial communities, shotgun metagenomic sequencing was conducted on the DNA from each tree sample and on composite DNA samples for each grass plot (n = 56). Sequencing was performed by Otago Genomics Ltd., New Zealand, on a NextSeq 2000 P3-300 instrument, producing sequence lengths of 2 x 150 bp.

### Quantification and statistical analysis

#### Bioinformatics and statistical analyses

The amplicon sequence data and downstream statistical analyses were processed in R version 4.3.0^72^ and Primer version 7.^73^ We removed the ITS data primers using the Cutadapt tool due to the sequence length variability of the ITS region.^74^ Demultiplexed sequences were processed following the DADA2 pipeline^75^ to classify reads into exact amplicon sequence variants (ASVs) at single-nucleotide level using the DADA2 algorithm. This involved trimming and filtering the sequence data to remove 16S primers, low-quality reads and chimaeras. We used the RDP’s naïve Bayesian classifier for taxonomic assignment.^76^ We used the SILVA rRNA gene database version 138.1^77^ to classify bacterial sequence data and the Unite database version 9^78^ to classify fungal sequence data. For the 16S rRNA data, we excluded ASVs not classified as bacterial, and those categorised as chloroplasts or mitochondria. For the ITS rRNA data we excluded ASVs not classified as fungi. For alpha diversity analyses, which are more sensitive to biases arising from sequencing depth, we used the *rarefy_even_depth* function in the ‘phyloseq’ package^79^ to randomly subsample the amplicon data without replacement to an even depth of 6500 and 11632 reads per sample for the bacterial and fungal data respectively. For all downstream beta-diversity analyses, we removed all samples with < 1000 reads, retaining 61 samples for the bacterial data and all 64 samples for the fungal data. We then performed Cumulative Sum Scaling (CSS) normalisation on the non-rarefied data.^80^ Phylogenetic trees were made by aligning the sequences using MAFFT (v 7.505)^81^ with default settings, then trimmed using trimA1 (v 1.2 rev 59)^82^ with parameters set to -gt 0.3 and -st 0.001. The bacterial and fungal trees were constructed using FastTree (v 2.1.10)^83^ with -gtr and -nt options selected. For the shotgun metagenomic data processing, we assessed the quality of the reads using FastQC (v0.11.7).^84^ Reads were trimmed using Trimmomatic (v 0.38)^85^ with a minimum Phred score of 10, and adapters were removed using ILLUMINACLIP. We used Prodigal (v.2.6.3)^86^ to predict open reading frames and translate the reads into protein sequences. Sequences were aligned against a protein reference database using DIAMOND (v2.1.6).^87^ We used MEGAN6^88^ to align the reads to genes with classified functional roles that were further categorised into curated SEED subsystems.^89^ We mapped the reads to KEGG Orthology (KO) groups using the KEGG database, release 105.^90^ We used the SEED assignments to analyse functional potential diversity and the KEGG assignments to identify functional groups and pathways representative of soil from monoculture or mixed plots. For the soil chemical data, we identified highly correlated variables (with a Pearson’s correlation value greater than 0.65 or less than -0.65) and selected one of the variables as representative for all downstream analyses. We retained nine of the sixteen soil chemical variables in our analyses (Table S3).

#### Quantitative analyses

To analyse the environmental profiles of the samples, we computed a Principal Coordinates Analysis (PCO) ordination based on a Euclidean distance matrix of the soil chemical variables. We mapped the first axis score to the GPS coordinates of each sample and plotted these onto a site map using QGIS version 3.28,^91^ with the base map sourced from the LINZ Data Service and licensed for reuse under the CC BY 4.0 license.

All comparative analyses between the monoculture and mixed plots data were undertaken on the soil samples from around mānuka trees, i.e., excluding samples collected from around other tree species in the mixed plots, unless stated otherwise. To analyse the alpha diversity of bacterial and fungal communities and their functional profiles from the different plot types (monoculture, mixed species and grass plots) and among each of the individual treatment plots (the six plots with trees), we computed Shannon diversity index (on the rarefied data) values using the *estimate_richness* function in the ‘phyloseq’ package.^79^ We assessed statistically significant differences in alpha diversity among the plot types and individual plots using a Dunn’s test. For the functional data, a Kruskal-Wallis test was used to test for significant differences in Shannon diversity between the monoculture and mixed plot samples; data from the grass plots were excluded for this analysis as we only had shotgun metagenomic sequence data for one representative grass sample per control plot, which was an insufficient number of independent samples for statistical analyses.

We examined differences in taxonomic composition and functional potential (based on ASVs or SEED subsystem level four assignments, respectively) among the plot types and individual plots by computing Bray-Curtis dissimilarity matrices using the ‘vegan’ package.^92^ We visualised the differences in composition using non-metric multidimensional scaling (nMDS). We tested for statistically significant differences in composition using a permutational multivariate analysis of variance (PERMANOVA) using the *adonis* function in the ‘vegan’ package.^92^ Procrustes rotation analyses were used to test the congruence between the bacterial and fungal data ordinations and the bacterial community compositional and functional ordinations, using the function *Procrustes* from the ‘vegan’ package, with permutational significance tests run using the function *protest* (based on 999 permutations).^92^ The first axis scores from the taxonomic and functional nMDS ordinations were extracted to visualise how the composition and functional potential of the communities changed across the individual treatment plots (monoculture and mixed). We investigated distance-decay relationships between the Bray-Curtis dissimilarity values of the communities under mānuka trees in the monoculture and mixed plots for both geographical distance (based on Euclidean distance of coordinates) and environmental distance (based on Euclidean distance of soil chemistry variables) using Mantel tests.

Additionally, we modelled the relationship among the individual soil chemical variables and the community composition and functional potential of the microbial communities to understand the relative importance of each variable. We applied distance-based multivariate multiple regression analyses using a forward selection procedure on each of the Bray-Curtis distance matrices using the ‘DistLM’ function in Primer version 7^73^ to examine the relationship between the soil chemical variables and microbial community composition and functional potential. We tested the significance of the relationships using 999 permutations. We used the *varpart.MEM* function^93^ to calculate the relative variance explained by the soil chemistry data and spatial factors (based on the coordinates of each sample) on each of the Bray-Curtis distance matrices. The soil chemistry data were standardized using the *decostand* function in the package ‘vegan’.^92^ Moran’s eigenvector maps (MEMs) were quantified using sample coordinates and those that significantly correlated with each of the Bray-Curtis matrices were used as the spatial components.

We identified KEGG Level 4 categories that were highly representative of the communities residing under the mānuka trees in the monoculture and mixed plots using an Indicator value (IndVal) analysis.^94^ We used Mann-Whitney U tests to compare the relative abundance of KEGG Level 2 groups within the metabolism category, KEGG Level 3 groups within the carbohydrate metabolism category and KEGG Level 4 groups within the energy metabolism category.

#### Quantification of community assembly processes

We used a two-step null model described by Stegen et al.,^95^^,^^96^ to evaluate the degree to which the bacterial and fungal communities in the monoculture and mixed plots are influenced by homogeneous selection, heterogeneous selection, homogenising dispersal, dispersal limitation and drift. Homogeneous selection refers to consistent environmental conditions being the primary cause of higher-than-expected similarity in community composition between local communities, while heterogeneous selection refers to variable environmental conditions leading to less similarity than expected in community composition.^97^^,^^98^ High rates of dispersal can lead to more similar composition than expected between local communities, even when abiotic conditions are distinct (homogenising dispersal). However, low dispersal rates between local communities can heighten the influence of ecological drift and lead to more dissimilar communities than otherwise expected (dispersal limitation), even when environmental conditions are similar.^97^^,^^98^

As illustrated in Figure 1, by Louisson et al.,^99^ the first step of the model involves using the *comdist* function from the package ‘picante’^100^ to calculate the observed phylogenetic turnover between each pair of local communities and compare this value to null model expectations, representing a β- nearest taxon index (βNTI) value.^97^ A βNTI value of <-2 or >+2 indicates that the community composition was significantly consistent with homogeneous or heterogeneous selection, respectively, as the assembly process. For the pairwise comparisons inconsistent with selection, the data were further partitioned into samples being dominated by homogenising dispersal, dispersal limitation, or those which were not dominated by selection or dispersal. Observed compositional turnover (based on Bray-Curtis dissimilarity) was calculated between each pair of local communities and compared to null model expectation (Raup-Crick-Bray; RCBray). The RCBray metric varies between -1 and +1, with a value <- 0.95 or >+0.95 specifying lower or higher than expected pairwise similarity in community composition than when drift is acting alone.^95^^,^^101^
