# Supplementary material for: Soil conditions are a more important determinant of microbial community composition and functional potential than neighboring plant diversity
Source: iScience. 2024 May 22;27(6):110056. doi: 10.1016/j.isci.2024.110056 (PMC11176639; doi:10.1016/j.isci.2024.110056)
Supplement: Document S1. Figures S1–S5 and Tables S1–S6 [file mmc1.pdf]

iScience, Volume *27*

Supplemental information

Soil conditions are a more important determinant of microbial community composition and functional potential than neighboring plant diversity

Ziva Louisson, Maria J. Gutiérrez-Ginés, Matthew Taylor, Hannah L. Buckley, Syrie M. Hermans, and Gavin Lear

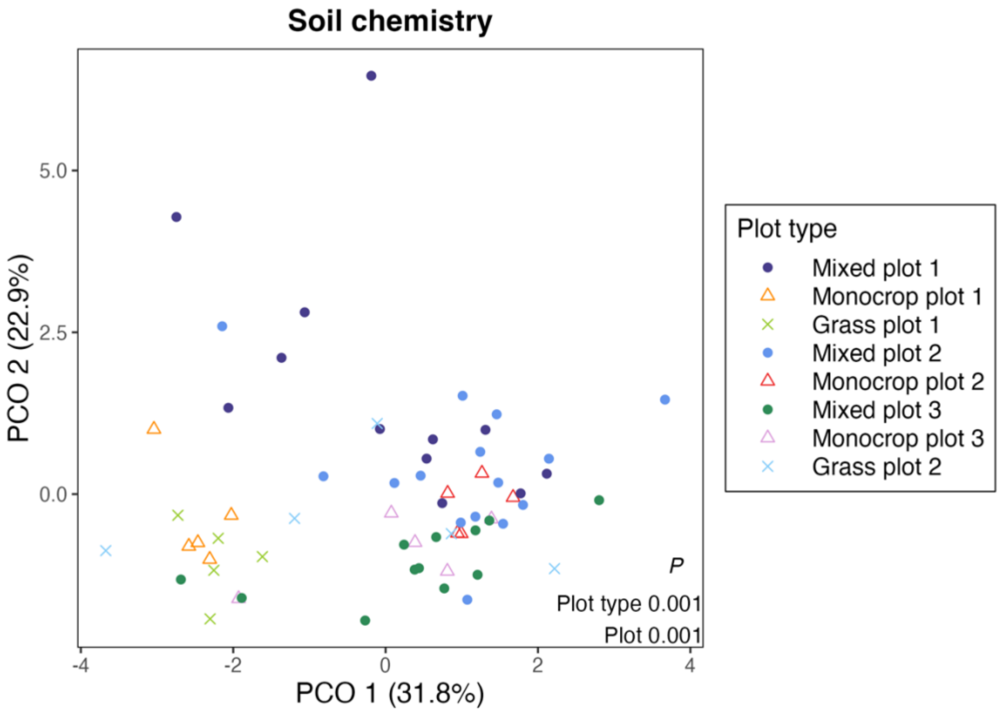

Figure S1 Soil chemistry profiles: Euclidean distance based principal coordinates analysis (PCO) of the soil chemistry profiles of each sample. *P*-values from PERMANOVAs assessing site-type effects are displayed in the bottom right of the plot. Related to Figure 1.

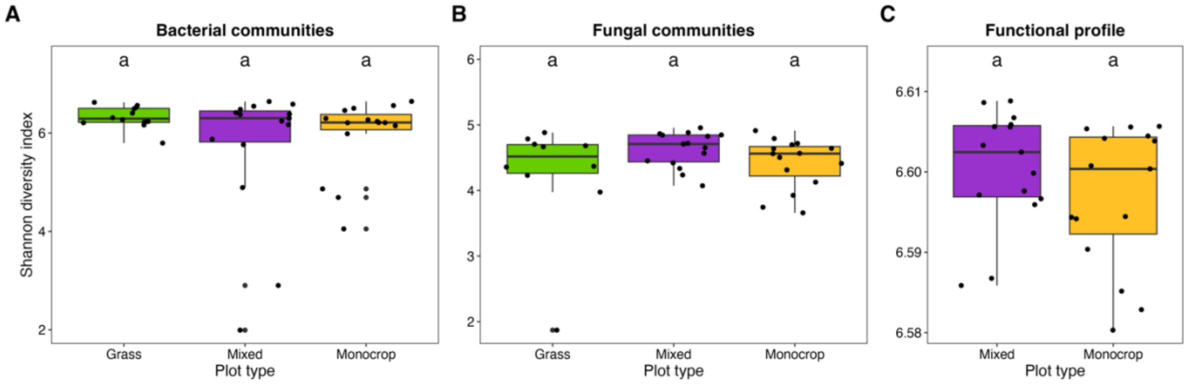

Figure S2 Alpha diversity for the different plot types: Boxplots displaying the estimated Shannon diversity index values of the **A** bacterial and **B** fungal communities and the **C** functional potential. The boxes represent the interquartile range (IQR: 25-75% of the data), the horizontal line indicates the median, while the whiskers extend to 1.5 times the IQR. The points represent individual values for each of the samples. Boxes with different letters within each panel indicate significant differences from each other ($P < 0.05$). Dunn's test was applied for the bacterial and fungal communities (**A-B)**, while a Kruskal-Wallis test was used to test for significant differences in the functional data (**C**). Related to Figure 2.

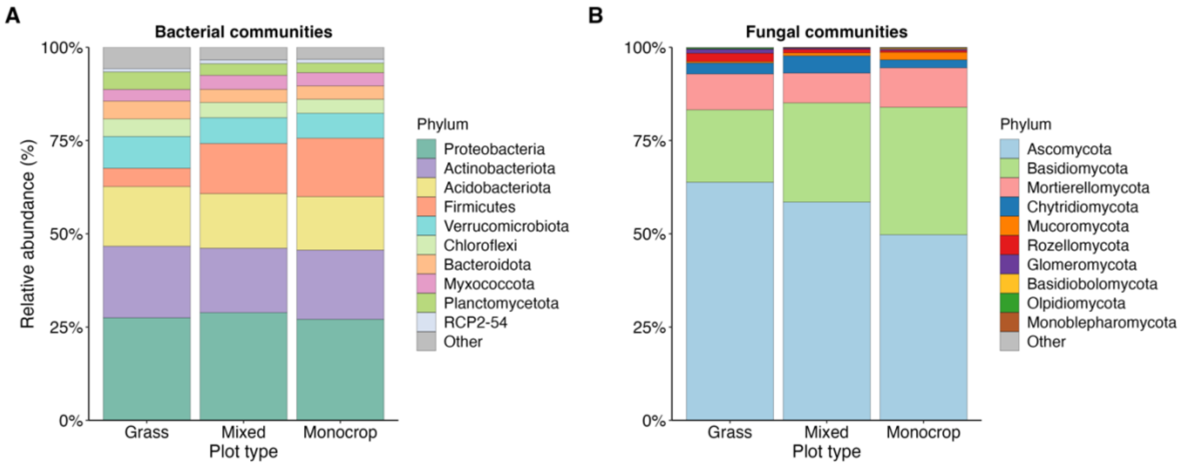

Figure S3 Stacked bar plots of the relative abundance of the top 10 most abundant phyla: Relative abundance in the **A** bacterial communities and **B** fungal communities

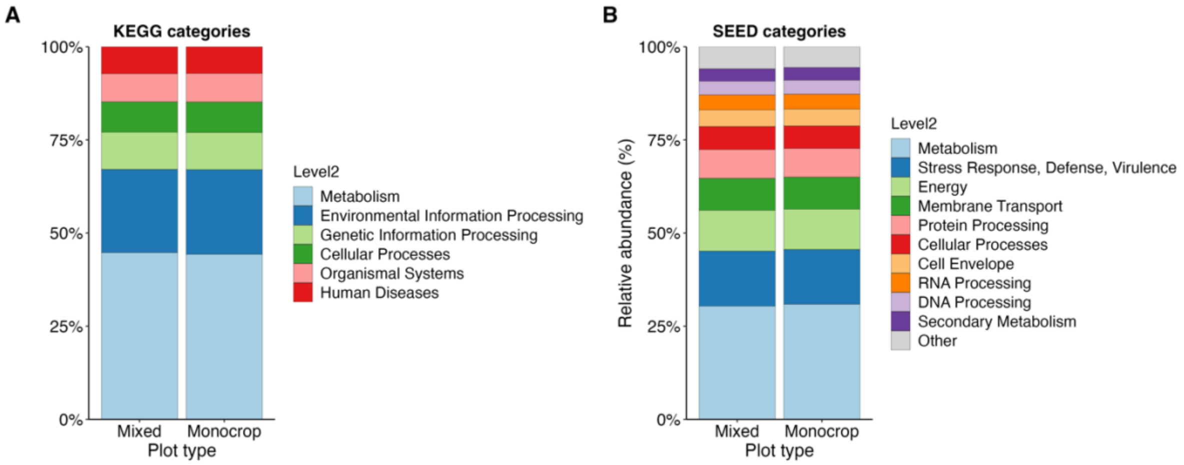

Figure S4 Stacked bar plots of the relative abundance of Level 1 functional categories: Relative abundance of the **A** Level 1 KEGG categories and **B** Level 1 SEED subsystems

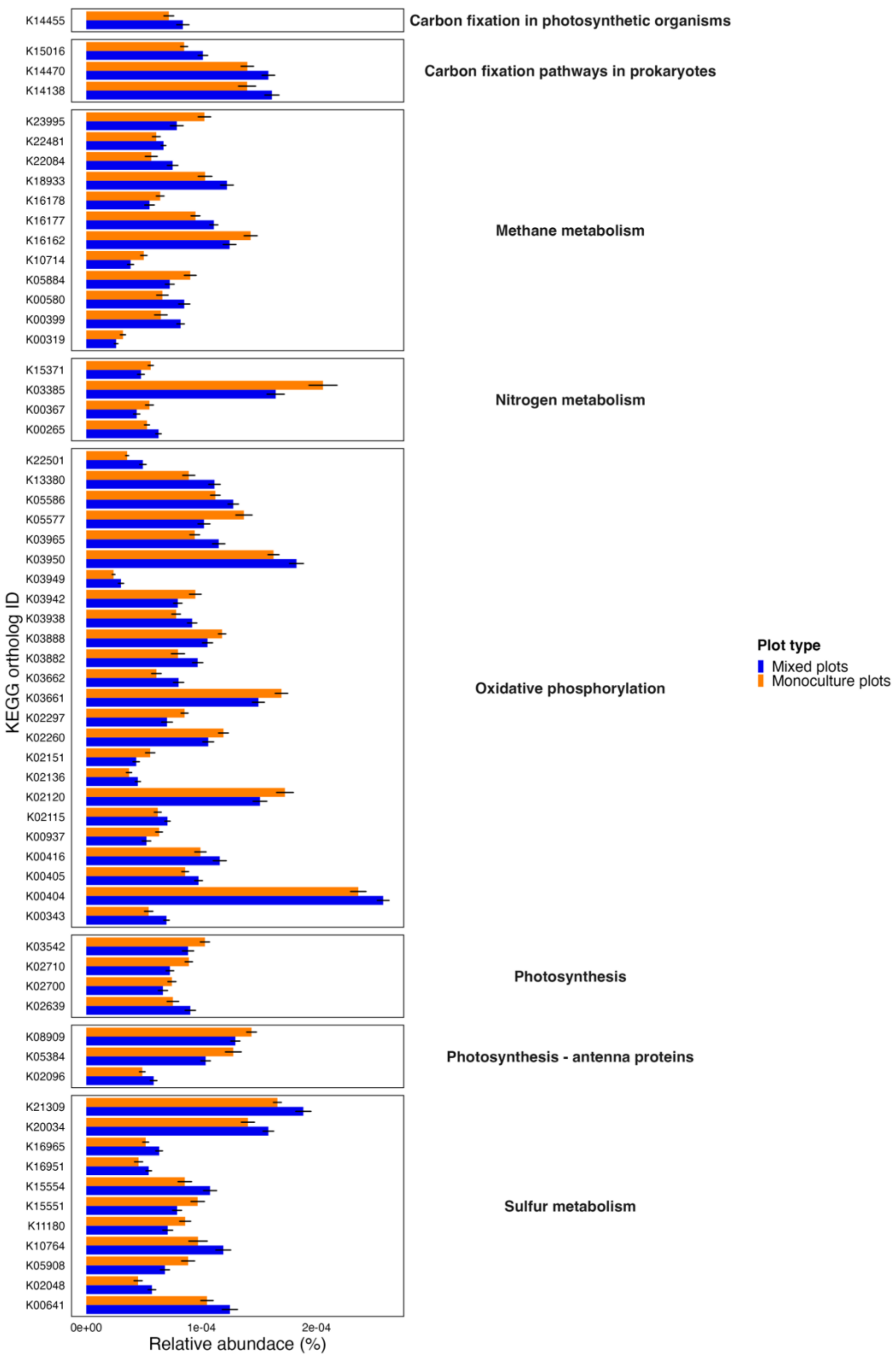

Figure S5 Abundance of Level 4 energy metabolism functional genes: Mean relative abundance of the Level 4 KEGG gene groups within the Level 2 energy metabolism category the significantly differed in relative abundance between the monoculture and mixed plots and split by the Level 3 KEGG categories. Related to Figure 5.

Table S1 List of plant species: Tree species planted in the mixed species plots as described by [1]. Plant names in bold are the additional eight tree species selected for reference.

Vernacular name	Scientific name
**Maanuka**	***Leptospermum scoparium***
**Harakeke**	***Phormium tenax***
**Ti koouka, cabbage tree**	***Cordyline australis***
**Karamuu**	***Coprosma robusta***
Mingimingi	*Coprosma propinqua*
Puurei makura	*Carex secta*
Swamp sedge	*Carex virgata*
Swamp coprosma	*Coprosma tenuicaulis*
Lake clubrush	*Schoenoplectus tabernaemontani*
Baumea	*Machaerina articulata*
**Koromiko**	***Veronica stricta***
Kaanuka	*Kunzea robusta*
Kahakitea	*Dacrycarpus dacrydioides*
Totara	*Podocarpus totara*
Toetoe	*Austroderia fulvida*
Round-leaved coprosma	*Coprosma rotundifolia*
**Akeake**	***Dodonaea viscosa***
**Kohukohu, Black matipo**	***Pittosporum tenuifolium***
**Tarata, lemonwood**	***Pittosporum eugenioides***
Red matipo	*Myrsine australis*
Kauri	*Agathis australis*

Table S2 Soil chemistry data: Mean values and standard deviations for each of the non-correlating soil chemical factors for each plot

Variable	Mixed plot 1	Monoculture plot 1	Grass plot 1	Mixed plot 2	Monoculture plot 2	Mixed plot 3	Monoculture plot 3	Grass plot 2
Water content (% dry wet)	26 ± 7.7	23.8 ± 4.9	22.4 ± 2.8	19.3 ± 4.1	19.2 ± 1.3	19.25 ± 1.4	17.6 ± 1.9	22.2 ± 1.5
pH	6.2 ± 0.2	7.2 ± 0.3	7.6 ± 0.2	6.2 ± 0.2	6.3 ± 0.1	6.5 ± 0.4	6.6 ± 0.7	6.6 ± 0.6
NH4-N (mg/kg)	9 ± 5.8	1.4 ± 0.9	2 ± 0	9.9 ± 9.6	11 ± 6	5.7 ± 6.4	6.4 ± 5.7	7.4 ± 6.6
C:N	11.2 ± 0.4	11.4 ± 0.5	11.2 ± 0.4	11.3 ± 0.5	12 ± 0	11.8 ± 0.5	12 ± 0	11.2 ± 0.4
NO3-N (mg/kg)	28.8 ± 11.9	41.4 ± 5.7	38 ± 6.6	23.8 ± 10.7	21.2 ± 3	23.2 ± 11.5	24.6 ± 8.3	29.6 ± 10.7
Olsen P (mg/kg)	62 ± 11.9	83.4 ± 9	73.6 ± 15.8	53.4 ± 10.4	59.4 ± 5.9	66.5 ± 10.7	72 ± 7.3	71 ± 35.5
Exchangeable Mg (cmol(+)/kg)	2.4 ± 0.7	1.6 ± 0.3	1.6 ± 0.2	2.1 ± 0.2	2.1 ± 0.2	1.8 ± 0.2	2.1 ± 0.5	2.2 ± 0.3
Exchangeable K (cmol(+)/kg)	0.4 ± 0.1	0.4 ± 0.1	0.5 ± 0.1	0.3 ± 0.1	0.5 ± 0.2	0.4 ± 0.1	0.7 ± 0.2	0.6 ± 0.2
Exchangeable Na (cmol(+)/kg)	0.3 ± 0.1	0.3 ± 0	0.2 ± 0.1	0.3 ± 0.1	0.3 ± 0	0.2 ± 0	0.3 ± 0	0.2 ± 0

Table S3 Explanatory variables used to explain sources of variation in bacterial community composition: Variables with the same letter (superscript) correlated with each other (Pearson's correlation >0.65 or <-0.65).

	**Soil**
**Variables included**	pH[a], Olsen phosphorous[b], NO3-N (mg/kg)[c], Water content (% dry wt)[d], NH4-N (mg/kg), Carbon to nitrogen ratio, Exchangeable K (cmol(+)/kg), Exchangeable Mg (cmol(+)/kg), Exchangeable Na (cmol(+)/kg)
**Variables omitted due to correlations**	Exchangeable Ca (cmol(+)/kg)[a], Base saturation (%)[a], Organic carbon (%)[b], Total nitrogen(%)[b], EC (dS/m)[c], Cation Exchange Capacity [c], Air dry soil water content (%)[d]

Table S4 Results of the pairwise PERMANOVA: Testing for the effect of plot type on bacterial community composition

	**Pairwise comp**	**d.f.**	**Sum of Sqs**	**R^2**	**F**	**P**
**Bacterial communities**	Grass x Monoculture	1	0.69	0.12	3.09	0.008
	Grass x Mixed	1	1.02	0.20	5.22	0.002
	Monoculture x Mixed	1	0.31	0.06	1.69	0.038
**Fungal communities**	Grass x Monoculture	1	0.73	0.12	3.17	0.001
	Grass x Mixed	1	0.70	0.12	3.23	0.001
	Monoculture x Mixed	1	0.47	0.08	2.27	0.003

Table S5 Results of the distance-based multivariate multiple regression analyses: Analysis conducted on the Bray-Curtis distance matrices of the bacterial, fungal and functional compositions.

	**Bacterial community composition**		**Fungal community composition**		**Functional composition (SEED)**	
**Variable**	**Proportion**	**P value**	**Proportion**	**P value**	**Proportion**	**P value**
pH	0.21	**0.001**	0.10	**0.001**	0.19	**0.001**
Water content	0.10	**0.005**	0.12	**0.001**	0.07	**0.009**
Exchangeable Mg	0.04	0.094	0.03	0.330	0.03	0.281
Olsen phosphorous	0.04	0.168	0.02	0.046	0.03	0.147
Exchangeable Na	0.03	0.231	0.03	0.353	0.03	0.212
Exchangeable K	0.03	0.376	0.05	**0.003**	0.03	0.532
Carbon to nitrogen	0.02	0.677	0.03	0.156	0.03	0.678
Nitrate	0.03	0.522	0.03	0.257	0.03	0.395
Ammonium	0.03	0.553	0.03	0.555	0.03	0.242

Table S6 Results of the variance partitioning: The amount of variation in bacterial, fungal and functional composition of the soil communities, based on variance partitioning (VarPart), that could be explained by space and the soil chemistry factors

	Soil chemistry	Spatial factors	Soil chemistry and spatial factors combined	Unexplained
**Bacterial communities**	11%	6%	16%	67%
**Fungal communities**	6%	5%	18%	72%
**Functional potential**	5%	4%	18%	73%

References

1. Gutiérrez-Ginés, M.J., Bisht, A., Meister, A., Robinson, B.H., Clarke, D., Tupuhi, G., et al. (2022) Maanuka dominated ecosystems to improve water and soil quality in Lake Waikare catchment, Waikato. ESR: Christchurch, New Zealand
